# Epigenome-wide gene–age interaction analysis reveals reversed effects of *PRODH* DNA methylation on survival between young and elderly early-stage NSCLC patients

**DOI:** 10.18632/aging.103284

**Published:** 2020-06-08

**Authors:** Chao Chen, Yongyue Wei, Liangmin Wei, Jiajin Chen, Xin Chen, Xuesi Dong, Jieyu He, Lijuan Lin, Ying Zhu, Hui Huang, Dongfang You, Linjing Lai, Sipeng Shen, Weiwei Duan, Li Su, Andrea Shafer, Thomas Fleischer, Maria Moksnes Bjaanæs, Anna Karlsson, Maria Planck, Rui Wang, Johan Staaf, Åslaug Helland, Manel Esteller, Ruyang Zhang, Feng Chen, David C. Christiani

**Affiliations:** 1 Department of Biostatistics, Center for Global Health, School of Public Health, Nanjing Medical University, Nanjing 211166, Jiangsu, China; 2 China International Cooperation Center for Environment and Human Health, Nanjing Medical University, Nanjing 211166, Jiangsu, China; 3 Department of Environmental Health, Harvard T.H. Chan School of Public Health, Boston, MA 02115, USA; 4 Department of Epidemiology and Biostatistics, School of Public Health, Southeast University, Nanjing 210009, Jiangsu, China; 5 Department of Bioinformatics, School of Biomedical Engineering and Informatics, Nanjing Medical University, Nanjing 211166, Jiangsu, China; 6 Pulmonary and Critical Care Division, Department of Medicine, Massachusetts General Hospital and Harvard Medical School, Boston, MA 02114, USA; 7 Department of Cancer Genetics, Institute for Cancer Research, Oslo University Hospital, Oslo 0424, Norway; 8 Division of Oncology and Pathology, Department of Clinical Sciences Lund and CREATE Health Strategic Center for Translational Cancer Research, Lund University, Lund 22381, Sweden; 9 Department of Medical Oncology, Jinling Hospital, School of Medicine, Nanjing University, Nanjing 210002, Jiangsu China; 10 Institute of Clinical Medicine, University of Oslo, Oslo 0424, Norway; 11 Josep Carreras Leukaemia Research Institute, Badalona, Barcelona, 08021, Catalonia, Spain; 12 Centro de Investigacion Biomedica en Red Cancer, Madrid 28029, Spain; 13 Institucio Catalana de Recerca i Estudis Avançats, Barcelona 08010, Catalonia, Spain; 14 Physiological Sciences Department, School of Medicine and Health Sciences, University of Barcelona, Barcelona 08007, Catalonia, Spain; 15 State Key Laboratory of Reproductive Medicine, Nanjing Medical University, Nanjing 211166, Jiangsu, China; 16 Jiangsu Key Lab of Cancer Biomarkers, Prevention and Treatment, Cancer Center, Collaborative Innovation Center for Cancer Personalized Medicine, Nanjing Medical University, Nanjing 211166, Jiangsu, China

**Keywords:** overall survival, DNA methylation, methylation–age interaction analysis, non-small cell lung cancer, aging

## Abstract

DNA methylation changes during aging, but it remains unclear whether the effect of DNA methylation on lung cancer survival varies with age. Such an effect could decrease prediction accuracy and treatment efficacy. We performed a methylation–age interaction analysis using 1,230 early-stage lung adenocarcinoma patients from five cohorts. A Cox proportional hazards model was used to investigate lung adenocarcinoma and squamous cell carcinoma patients for methylation–age interactions, which were further confirmed in a validation phase. We identified one adenocarcinoma-specific CpG probe, cg14326354_*PRODH*_, with effects significantly modified by age (*HR*_interaction_ = 0.989; 95% CI: 0.986–0.994; *P* = 9.18×10–7). The effect of low methylation was reversed for young and elderly patients categorized by the boundary of 95% CI standard (*HR*_young_ = 2.44; 95% CI: 1.26–4.72; *P* = 8.34×10-3; *HR*_elderly_ = 0.58; 95% CI: 0.42–0.82; *P* = 1.67×10-3). Moreover, there was an antagonistic interaction between low cg14326354_*PRODH*_ methylation and elderly age (*HR*_interaction_ = 0.21; 95% CI: 0.11–0.40; *P* = 2.20×10−6). In summary, low methylation of cg14326354PRODH might benefit survival of elderly lung adenocarcinoma patients, providing new insight to age-specific prediction and potential drug targeting.

## INTRODUCTION

Population aging has resulted in a rapid increase in lung cancer cases as well as corresponding surgeries among elderly patients [1]. Indeed, the median age at diagnosis of lung cancer is 70 years old [2]. Further, lung cancer leads as a cause of cancer deaths among men ≥40 years old and women ≥60 years old [3].

Progression of lung cancer is, in part, due to accumulation of genomic instability as well as age-related declines in system integrity and function [4]. Thus, even for individuals exposed to similar levels of risk factors, lung cancer severity can vary as a function of individual differences in aging. Therefore, compared to predictive guidance for the overall population, effective predictive guidance for age-specific populations, especially elderly patients, is needed to better guide postoperative treatment and improve survival. Developing such guidance necessitates identifying exclusive prognostic indicators of lung cancer for the elderly.

Epigenetic mechanisms represent the molecular interface mediating gene–environment interactions throughout the lifecycle [5]. DNA methylation, a reversible epigenetic modification, correlates with tumor prognosis in almost all cancers including non-small cell lung cancer (NSCLC) [6–9]. DNA methylation events may potentially be cancer biomarkers as well as therapeutic targets to improve cancer treatment [10].

Alterations to DNA methylation often occur during aging [11]. One of these alterations, known as “epigenetic drift”, may further contribute to tumorigenesis in the elderly [12]. Changes in DNA methylation also can contribute to senescence [13]. However, it remains largely unclear whether alterations of methylation patterns resulting from aging, accumulating environmental exposures throughout life [14], and other events also have varied effects on cancer survival during aging. Such phenomena may further explain the increased alteration of cancer mortality risk with age and may increase the effectiveness of cancer prediction and treatment.

We hypothesized that the methylation effect on cancer survival changes during aging. Thus, identifying age-specific signatures will be critical for prognosis prediction, underpinning potential preventative strategies, and improving survival for elderly patients. However, most epigenome-wide association studies are designed to identify main effects of DNA methylation and fail to provide knowledge about changes in epigenetic effects during aging. Thus, we performed an epigenome-wide methylation–age interaction analysis to identify age-specific, prognosis-associated epigenetic biomarkers using NSCLC patients from four cohorts, along with an independent population from The Cancer Genome Atlas (TCGA) to confirm our results.

## RESULTS

After quality control (QC) procedures, 1,230 lung adenocarcinoma (LUAD) and lung squamous cell carcinoma (LUSC) patients with 311,891 CpG probes remained for subsequent association analysis. There were 613 (*N*_LUAD_ = 492; *N*_LUSC_ = 121) patients in the discovery phase, and 617 (*N*_LUAD_ = 332; *N*_LUSC_ = 285) patients in the validation phase. The average age was 66.4 and 66.5 years for patients in the discovery and validation phases, respectively. Most NSCLC patients were in stage I (77.5% in discovery; 63.7% in validation) (Supplementary Table 1).

**Figure 1:**
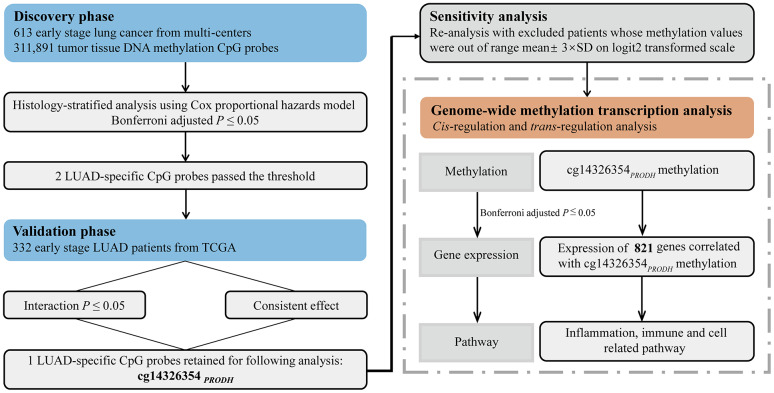
**Flow chart of study design and statistical analyses.**

We only observed two significant methylation–age interactions for LUAD patients in the discovery phase (Figure 1, Supplementary Figure 1, Supplementary Table 2), and none for LUSC patients. Results of the epigenome-wide association study are shown in Supplementary Materials 1 and 2. In the validation phase, only one LUAD-specific CpG probe, located in proline dehydrogenase 1 (*PRODH*) (Supplementary Table 3), remained significant.

Low methylation of cg14326354_*PRODH*_ interacted with age to affect survival of patients (discovery phase: hazard ratio (HR)_interaction_ = 0.982; 95% CI: 0.976–0.989; *P* = 1.11×10^–7^; validation phase: HR_interaction_ = 0.981; 95% CI: 0.966–0.997; *P* = 0.0202; combined data: HR_interaction_ = 0.989; 95% CI: 0.986–0.994; *P* = 9.18×10^–7^). Further, the robustly significant interaction effect was confirmed in sensitivity analysis by removing outliers in methylation data (Supplementary Table 4). When using leave-one-out method for validation, the interaction remained significant (Supplementary Figure 2). Moreover, meta-analysis also exhibited significant (HR_interaction_ = 0.983; 95% CI: 0.976–0.990; *P* = 3.95×10^–6^) and homogenous (*P*_Heterogeneity_ = 0.97) interaction effects across five cohorts (Supplementary Figure 3). Based on stratified analysis by smoking status, sex, clinical stage, and study cohort, there was no significant heterogeneity of interaction effect between subgroups of any of these covariates (Supplementary Table 5).

**Figure 2:**
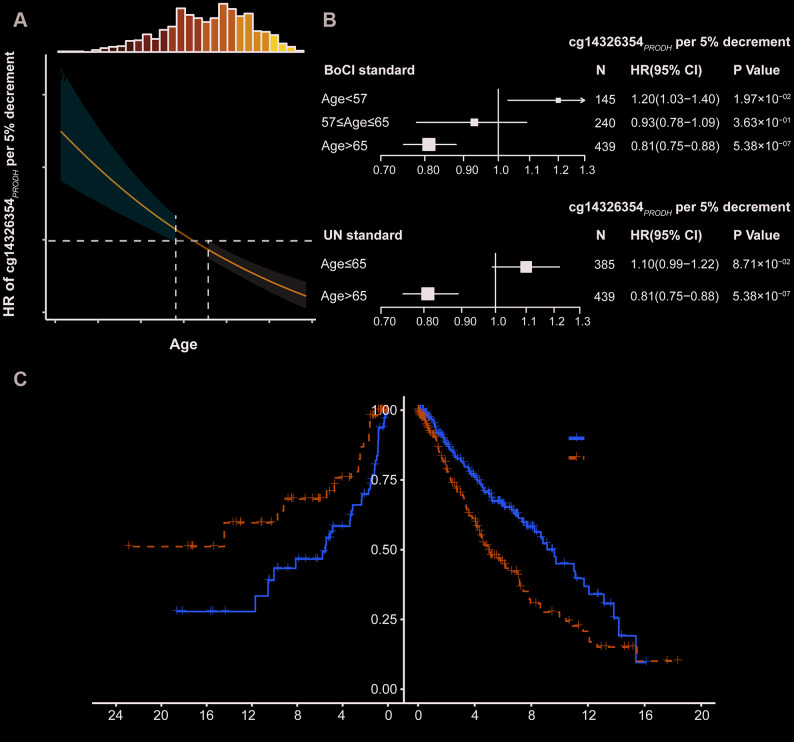
**DNA methylation and age interaction on survival of lung adenocarcinoma (LUAD) patients.** (**A**) Hazard ratio (HR) of cg14326354_*PRODH*_ 5% per decrement of methylation level among different aged patients. The 95% confidence interval (95% CI) band of HR for patients aged <57 or >65 years is statistically significant. Top histogram shows distribution of age. (**B**) Forest plots of HR of cg14326354_*PRODH*_ 5% per decrement of methylation level in young and elderly LUAD patients, categorized based on boundary of 95% CI (BoCI) and 1956 United Nations standard. (**C**) Kaplan-Meier survival curves of low and high methylation groups (categorized by median value) among young and elderly LUAD patients defined using BoCI standard. *P*_heterogeneity_ was used to evaluate heterogeneity of HRs across age groups.

With increased age, there was an increased protective effect for low methylation of cg14326354_*PRODH*_ on LUAD survival (Figure 2A, Supplementary Figure 4). Thus, age was a modifier of the association between cg14326354_*PRODH*_ and survival. To better understand the interaction between DNA methylation and age, patients were categorized into young and elderly groups based on the boundary of 95% CI (BoCI) of HR (<57 vs >65 years in Figure 2A) or the United Nations (UN) standard (≤65 vs >65 years). The BoCI standard provided stable results in both phases as well as combined data (Supplementary Table 6), with varied effects of cg14326354_*PRODH*_ methylation across different age groups. Low methylation of cg14326354_*PRODH*_ showed a risk effect on survival for young patients (HR_BoCI_ = 1.20; 95% CI: 1.03–1.40; *P* = 1.97×10^–2^; HR_UN_ = 1.10; 95% CI: 0.99–1.22; *P* = 8.71×10^–2^) but benefited survival of elderly LUAD patients (HR_BoCI_ = 0.81; 95% CI: 0.75–0.88; *P* = 5.38×10^–7^; HR_UN_ = 0.81; 95% CI: 0.75–0.88; *P* = 5.38×10^–7^) (Figure 2B). Kaplan-Meier curves also confirmed reversed effect patterns across age groups based on BoCI standard (HR_young_ = 2.44; 95% CI: 1.26–4.72; *P* = 8.34×10^-3^; HR_elderly_ = 0.58; 95% CI: 0.42–0.82; *P* = 1.67×10^-3^), with methylation groups defined by median values. There was significant heterogeneity of the low cg14326354_*PRODH*_ methylation effect between young and elderly patients (*I^2^* = 93.03%, *Q* = 14.35, *P* = 1.52×10^-4^) (Figure 2C). These results indicated that elderly LUAD patients had better survival with lower methylation of cg14326354_*PRODH*_.

In addition, we evaluated the joint effect of cg14326354_*PRODH*_ methylation level (low vs high) and age (elderly vs young) on LUAD survival (Table 1). The group with the best survival (young patients with high methylation) was used as the reference to evaluate effects of low methylation, elderly age, and their interaction. The main effect of low cg14326354_*PRODH*_ methylation was HR = 2.84 (95% CI: 1.59–5.08, *P* = 4.35×10^−4^), and the main effect of elderly age was HR = 3.18 (95% CI: 1.85–5.46, *P* = 2.64×10^−5^). However, the joint effect was HR = 1.86 (95% CI: 1.08–3.19, *P* = 2.42×10^−2^), which was less than the product of the two main effects (2.84×3.18 = 9.03). This result indicates an antagonistic interaction between low cg14326354_*PRODH*_ methylation and elderly age (HR_interaction_ = 0.21; 95% CI: 0.11–0.40; *P* = 2.20×10^−6^).

**Table 1:** Joint effect and interaction of low methylation and elderly age on the prognosis of early-stage lung adenocarcinoma (LUAD).

**Effect type ^a^**	**Elderly ^b^**	**Low methylation**	**Number**	**Death**	**Crude mortality**	**HR (95% CI) ^a^**	***P*^a^**
	No	No	75	17	22.67%	Ref.	
Main effect _1_	No	Yes	70	33	47.14%	2.8398 (1.5876,5.0798)	4.35×10^-4^
Main effect _2_	Yes	No	217	98	45.16%	3.1804 (1.8542,5.4553)	2.64×10^-5^
Joint effect	Yes	Yes	222	70	31.53%	1.8590 (1.0840,3.1890)	0.0242
Interaction ^c^						0.2058 (0.1070,0.3961)	2.20×10^-6^

^a^ Patients categorized into two groups (low vs high) by medium of cg14326354_*PRODH*_ methylation level. Classification criteria of age were based on boundary of 95% confidence interval (CI) standard (young: <57 years; elderly >65 years).

^b^ Main effects of low methylation and elderly age and their joint effect and interaction were derived from Cox proportional hazards model adjusted for covariates.

^c^ Interaction = Joint effect ÷ (Main effect _1_ × Main effect _2_). 0.2058 ≈ 1.8590 ÷ (3.1804 × 2.8398).

**Figure 3:**
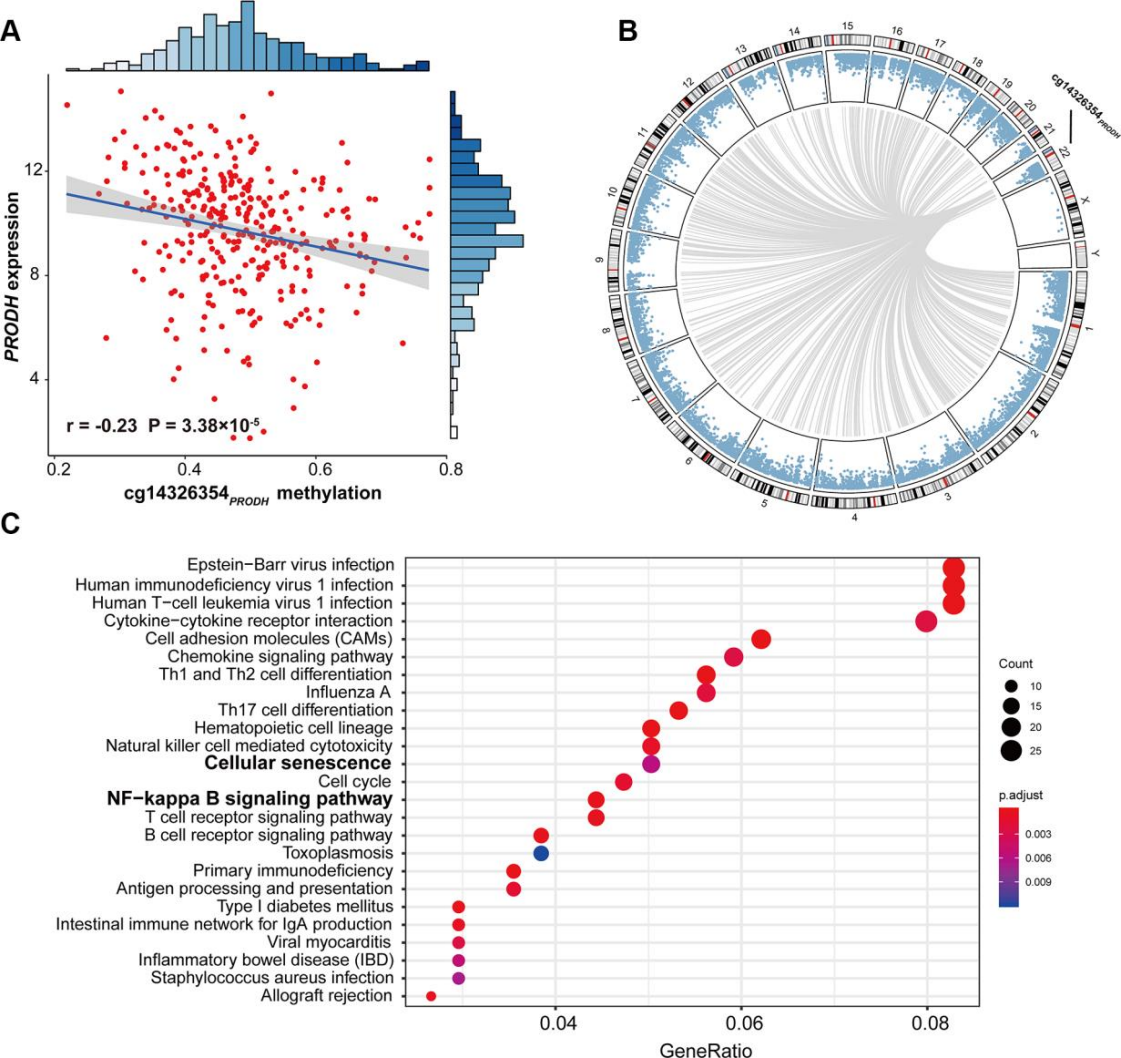
**Scatter plot of *cis*-regulation, circos plot of genome-wide *trans*-regulation analysis, and significant pathways of gene enrichment pathway analysis.** (**A**) Correlation between DNA methylation of cg14326354_*PRODH*_ and expression of *PRODH*. The *r* coefficient and *P-*value were derived from Pearson correlation analysis. Gene expression was log2-transformed before correlation analysis. (**B**) Circos plot of genome-wide *trans*-regulation analysis in the TCGA cohort. Blue points ordered by genomic position represent *P*-values of correlation between gene expression and methylation at cg14326354_*PRODH*_. Grey lines represent significant correlations with Bonferroni-adjusted *P* ≤ 0.05. (**C**) KEGG gene enrichment analysis of 821 *trans*-regulated genes correlated with cg14326354_*PRODH*_ methylation.

Further, *cis*-regulation and genome-wide *trans*-regulation analyses were conducted in the TCGA cohort. We observed significant correlation between cg14326354_*PRODH*_ and *PRODH* expression (*r* = –0.23; *P* = 3.38 × 10^-5^) in LUAD patients (Figure 3A), indicating that cg14326354_*PRODH*_ *cis*-regulated gene expression. Moreover, genome-wide *trans*-regulation analysis revealed that expression of 821 genes was significantly correlated with methylation level of cg14326354_*PRODH*_ (Supplementary Material 3, Figure 3B). KEGG enrichment analysis based on 821 *trans*-regulated genes showed several significant immune- or inflammation-related pathways, such as chemokine signaling, T cell receptor and B cell receptor signaling, and cellular pathways such as cell differentiation and cell cycle (Figure 3C). Notably, these *trans*-regulated genes were also enriched in senescence-related pathways (e.g., *cellular senescence*) and cancer-related pathways (e.g., *NF-κB signaling*).

Because tumor mutational burden (TMB) serves as a biomarker to select patients who might benefit from immune checkpoint inhibitors [15], we also evaluated association between TMB and cg14326354_*PRODH*_ as well as *PRODH* expression. TMB of each sample is shown in Supplementary Material 4. cg14326354_*PRODH*_ was positively correlated with TMB (*r* = 0.23; *P* = 4.04×10^-5^), while *PRODH* expression was negatively correlated with TMB (*r* = –0.22; *P* = 6.62×10^-5^) (Supplementary Figure 5).

## DISCUSSION

In this two-stage study using five independent cohorts, we systematically investigated methylation–age interactions on an epigenome-wide scale. Our results show an antagonistic interaction between elderly age and low methylation of cg14326354_*PRODH*_, indicating opportunities for epi-drug intervention due to the inherent reversibility of epigenetic events [16] and increasing treatment efficiency based on age-specific drug targeting.

*PRODH* is located in chromosome 22q11.2, a region often deleted in various human tumors. This gene encodes a mitochondrial inner membrane-associated enzyme that acts as a tumor suppressor in vitro and in vivo [17]. However, *PRODH* plays a paradoxical role in tumors. Hypoxia and nutrient depletion are important characteristics of the tumor microenvironment, where PRODH may serve as a tumor survival factor [18]. Indeed, *PRODH* supports tumor metastasis formation, and inhibiting its activity impairs cancer cell growth, indicating *PRODH* is a potential drug target [19]. A metabolic enzyme, *PRODH* can catabolize proline to pyrroline-5-carboxylate (P5C). The process can donate electrons that enter the electron transport chain to produce reactive oxygen species (ROS), which then participate in protective autophagy rather than apoptotic cell death [18].

**Figure 4:**
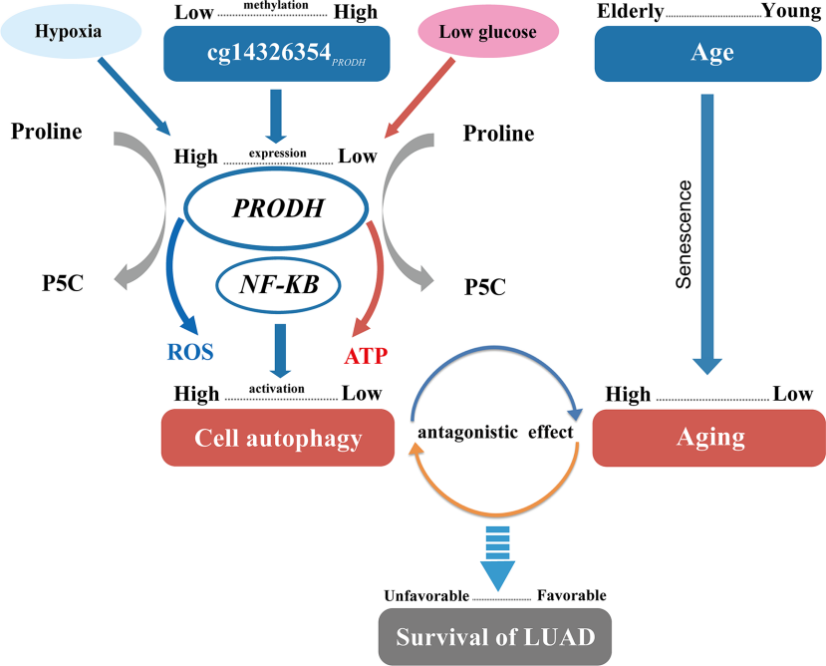
**Pathway of DNA methylation–age interaction effect on survival of lung adenocarcinomas (LUAD) patients.**

Autophagy, a self-digestion process, plays an important role in maintaining intracellular homeostasis. Autophagy can clear intracellular abnormally folded protein and dysfunctional organelles, inhibit cell stress response, and prevent genetic damage in early phases of tumorigenesis. However, autophagy helps tumor cells survive nutritional deficiencies and hypoxic conditions when tumors develop and accumulate more mutations to promote malignant progression [20]. Further, tumor-surrounding normal cells, which are active and essential parts of the microenvironment, support tumor proliferation by autophagy. Besides, autophagy in distant organs may also support growth of tumor tissue [21]. Additionally, autophagy can act as a mechanism of tumor resistance to chemotherapy agents and lead to antagonistic effects of gefitinib combined with cisplatin in NSCLC treatment, which may contribute to poor therapeutic effectiveness and patient prognosis [22, 23]. Further, our results suggest that low methylation of cg14326354_*PRODH*_ may potentially promote *PRODH* expression, further heighten autophagy to some extent [24], and then result in poor prognosis (Figure 4).

Age is an independent risk factor for lung cancer survival [25]. Individual aging implies a higher abundance of senescent cells in aged tissues and reflects an increase in the generation of senescent cells [26]. At old age, senescent cells generate a pro-tumorigenic microenvironment, though at young age these cells may protect against transformation into primary tumors [27]. A previous study also shows that p53 function declines during aging [28] and might promote tumor growth and decrease cancer survival [29]. Moreover, senescent cells can promote reprogramming of tumor stem cells, increase cancer stemness, and accelerate tumor growth [30]. Thus, combined with our results, increased generation of senescent may be relevant to poor NSCLC prognosis for elderly patients (Figure 4).

Autophagy is reduced in aging, likely through several mechanisms [31]. Lipofuscin produced during aging can destroy the function of lysosomes, restricting binding between autophages and lysosomes [32]. In addition, expression of lysosome-associated membrane glycoprotein (*LAMP2a*), which assists autophagy, decreases during aging and thus can inhibit autophagy [33]. Further, by guaranteeing stability of the cellular proteome and proper organelle turnover, autophagy can prevent or slow down aging and extend lifespan [34]. The antagonistic effect exists between aging and the autophagy level resulting from low methylation of cg14326354_*PRODH*_, in spite of the harmful effect of both, which could provide a possible mechanism of the cg14326354_*PRODH*_–age interaction (Figure 4).

The 821 significant *trans*-regulated genes we identified were enriched in KEGG pathways including inflammation and immune-related pathways. Notably, *cellular senescence* was involved in these pathways, again indicting potential indirect induction of cg14326354_*PRODH*_ on senescence. Meanwhile, the *NF-κB pathway*, with the ability to upregulate genes responsible for inflammation, cell survival, proliferation, invasion, angiogenesis, and metastasis, often plays a critical role in initiation, promotion, progression, and therapy resistance of cancers [35, 36]. Further, NF-κB family members can activate or inhibit signaling pathways, leading to induction of autophagy or transcription of certain pro-autophagic-regulating genes [35], and can induce senescence [37]. Because cell proliferation can be associated with both senescence and survival [38, 39], we also analyzed several proliferation-associated genes retrieved from the KEGG database. Expression of these genes were significantly correlated with cg14326354_*PRODH*_ methylation and affected LUAD survival, including *MKI67*, *BTG2*, *KIAA1524,* and *CDC123* (Supplementary Table 7). Our previous study of *BTG2* expression and methylation already indicated it is a prognostic biomarker of NSCLC [7]. These results also indicate the potential role of cg14326354_*PRODH*_ in indirect induction of autophagy, senescence, and survival. Further functional studies are warranted to elucidate the mechanism of cg14326354_*PRODH*_ and age interaction on LUAD survival.

Age represents a complex surrogate for a host of underlying phenomena, although its measurement is simple and accurate [40]. A previous study suggested that gene–age interactions may partially be surrogates for gene–gene and gene–environment interactions [41]. In a study investigating the efficacy of metronomic vinorelbine to treat patients with advanced unresectable NSCLC, age was an important factor that decreased treatment efficacy [42]. Our study might provide a novel explanation of age effects on treatment efficacy from the cg14326354_*PRODH*_–age interaction perspective. Further clinical studies will provide additional insight into cg14326354_*PRODH*_ and its age-specific effects in tumors, which may lead to new age-specific biomarkers and therapeutic strategies that improve prediction accuracy and treatment efficacy.

Our study has several strengths. First, this is the first epigenome-wide study to investigate the interaction between DNA methylation and age on NSCLC survival, which provides new evidence to account for the missing heritability of complex diseases [43] and may further reveal the role of age in heterogeneity of NSCLC prognosis and treatment efficacy. Second, to identify stable and reliable biomarkers, a two-stage study design along with Bonferroni correction and sensitivity analysis was used to exhaustively search for interactions, which is quite conservative in controlling for false positives. Finally, with a large sample size to analyze DNA methylation–age interactions, our study has improved statistical power to identify complex associations with small–medium effect size.

Nonetheless, several limitations also need to be acknowledged. First, we did not observe robustly significant methylation–age interactions on survival for LUSC, which may be due to limited sample size and thus insufficient power. However, there was no significant heterogeneous effect between LUAD and LUSC groups (Supplementary Table 8). Second, the association was no longer significant in young LUAD patients when the analysis used UN standards to define age groups. However, we still observed a significant association in patients <57 years old. This effect might be because >62% (240/385) of young patients defined using the UN standard (57–65 years) attenuated the effect of cg14326354_*PRODH*_ methylation. Therefore, high methylation of cg14326354_*PRODH*_ might benefit survival of young LUAD patients. Third, although widespread methylation–age interactions may exist, we only identified one interaction, which may be due to the most conservative correction method used in the discovery phase and limited statistical power in the validation phase due to low event rate of survival time in the TCGA population. We may need longer time to follow-up early-stage patients in TCGA for their events to occur. Nevertheless, the interaction between cg14326354_*PRODH*_ and age was successfully confirmed, indicating it was a conservative and robust association. Fourth, our analysis was based on the assumption of linear additive interaction, and new statistical models can be used to properly capture non-linear methylation–age interactions. Last, the *cis*-regulation pattern of cg14326354_*PRODH*_ requires more biological evidence, although methylation is believed to play a crucial role in regulating gene expression [44] and further influence disease gene function [45]. Therefore, our findings should be interpreted with caution, and functional experiments are warranted to confirm these associations.

In conclusion, low methylation of cg14326354_*PRODH*_ benefited survival of elderly LUAD patients. Our results have implications for not only age-specific prediction of cancer survival, but also possible methylation-specific drug targeting.

## MATERIALS AND METHODS

### Lung cancer study populations

Only early-stage (stage I–II) LUAD and LUSC patients were included in our study. DNA methylation data was harmonized from five cohorts: Harvard, Spain, Norway, Sweden, and TCGA.

### Harvard

Since 1992, patients have been recruited at Massachusetts General Hospital (MGH) and histologically confirmed as primary NSCLC [46]. We profiled 151 early-stage patients from this cohort. During curative surgery, tumor specimens were collected with complete resection and snap-frozen. A MGH pathologist evaluated each specimen for tumor cell amount (tumor cellularity > 70%) and quality. Specimens were classified histologically according to World Health Organization (WHO) criteria. The Institutional Review Boards at Harvard T.H. Chan School of Public Health and MGH approved the study. All patients provided written informed consent.

### Spain

The Spain cohort included 226 early-stage NSCLC patients recruited from eight sub-centers in 1991–2009 [47]. Tumor DNA was extracted from fresh-frozen tumor specimens and further checked for integrity and quantity. Patients provided written consent, and tumors were surgically collected. The study was approved by the Bellvitge Biomedical Research Institute Institutional Review Boards.

### Norway

The Norway study population consisted of 133 early-stage NSCLC patients from Oslo University Hospital-Riks Hospitalet, Norway, in 2006–2011 [48]. Tumor tissues were snap-frozen in liquid nitrogen and stored at –80°C until DNA isolation. The project was developed with approval of the Oslo University Institutional Review Board and Regional Ethics Committee (S-05307). All patients provided informed consent.

### Sweden

Tumor DNA was collected from 103 early-stage NSCLC patients, including 80 LUAD and 23 LUSC patients, at the Skane University Hospital in Lund, Sweden [49]. The study was developed under the approval of the Regional Ethical Review Board in Lund, Sweden (registration no. 2004/762 and 2008/702). All patients provided written informed consent.

### TCGA

A total of 332 LUAD and 285 LUSC cases with full DNA methylation, survival time, and covariate data were included. Level-1 HumanMethylation450 DNA methylation data of early-stage NSCLC patient were downloaded on October 01, 2015.

### Quality control for DNA methylation data

DNA methylation was assessed using Illumina Infinium HumanMethylation450 BeadChips (Illumina Inc.). Raw image data were imported into Genome Studio Methylation ModuleV1.8 (Illumina Inc.) to calculate methylation signals and to perform normalization, background subtraction, and QC. Unqualified probes were excluded if they fit any of the following criteria: (i) failed detection (*P* > 0.05) in ≥5% samples, (ii) coefficient of variance (CV) <5%, (iii) all samples methylated or all unmethylated, (iv) common single nucleotide polymorphisms located in probe sequence or in 10-bp flanking regions, (v) cross-reactive probes [50], or (vi) data did not pass QC in all cohorts. Samples with >5% undetectable probes were excluded. Methylation signals were further processed for quantile normalization (*betaqn* function in R package *minfi* [51]), type I and II probe correction (*BMIQ* function in R package *lumi* [52]), and adjusted for batch effects (*ComBat* function in R package *sva*) [53]. Details of QC process are described Supplementary Figure 6.

### Quality control for gene expression data

The TCGA workgroup completed mRNA sequencing data processing and QC. Raw counts were normalized using RNA-sequencing by expectation maximization (RSEM). Level-3 gene quantification data were downloaded from the TCGA data portal and were further checked for quality. Expression of genes was extracted and log2-transformed before analysis. Normalization results were then evaluated through boxplots of the distribution of gene expression across all samples (Supplementary Figure 7).

### Statistical analysis

Statistical analysis flow is presented in Figure 1. Patients from Harvard, Spain, Norway, and Sweden study cohorts were assigned to the discovery phase, while patients in TCGA were assigned to the validation phase.

In the discovery phase, histology-stratified analysis and Cox proportional hazards model adjusted for age, smoking status, sex, clinical stage, and study center were applied to test the methylation–age interaction effect on overall survival in LUAD and LUSC patients using the R package *survival* [54]. Hazard ratio (HR) and 95% confidence interval (CI) were described per 5% level of methylation decrement. The *P*-value threshold for multiple testing was established using the Bonferroni method, which set the significance level to 0.05 divided by number of tests. This way, overall type I error was controlled at the 0.05 level. In our study, significance level of interaction analysis was defined as 1.60×10^-07^ = 0.05/311,891. Interactions with *P* ≤ 1.60×10^-07^ were screened out and then further confirmed in the validation phase. Robustly significant probes were retained if they fit both of the following criteria: (i) interaction *P* ≤ 0.05, and (ii) direction of interaction effects was consistent across both phases. In sensitivity analysis, patients were excluded if their methylation values were out of range mean ± 3×standard deviation (SD) on logit_2_-transformed scale. Genome-wide expression correlation analysis was performed to identify potential *trans*-regulation genes in TCGA. KEGG enrichment analysis of potential *trans*-regulation genes (Bonferroni adjusted *P* < 0.05) was conducted using R package *clusterProfiler* [55].

Continuous variables were summarized as mean ± SD; categorical variables were described as n (%). Kaplan-Meier survival curves were used to illustrate survival difference between patients in low and high methylation groups (categorized by median value). We used two classification criteria to define young and elderly patients: (i) the UN standard (1956) of 65 years old as the cut-off value to distinguish elderly and young people [56], (ii) and a cut-off value calculated based on BoCI of HR of the CpG probe. All statistical analyses were performed in R version 3.5.1 (The R Foundation).

## Supplementary Material

Supplementary Figures

Supplementary Tables

Supplementary Material 1

Supplementary Material 2

Supplementary Material 3

Supplementary Material 4
