# Supplementary material for: Epigenome-wide gene–age interaction analysis reveals reversed effects of PRODH DNA methylation on survival between young and elderly early-stage NSCLC patients
Source: Aging (Albany NY). 2020 Jun 8;12(11):10642–62. doi: 10.18632/aging.103284 (PMC7346054; doi:10.18632/aging.103284)
Supplement: Supplementary Figures [file aging-12-103284-s002..pdf]

SUPPLEMENTARY FIGURES

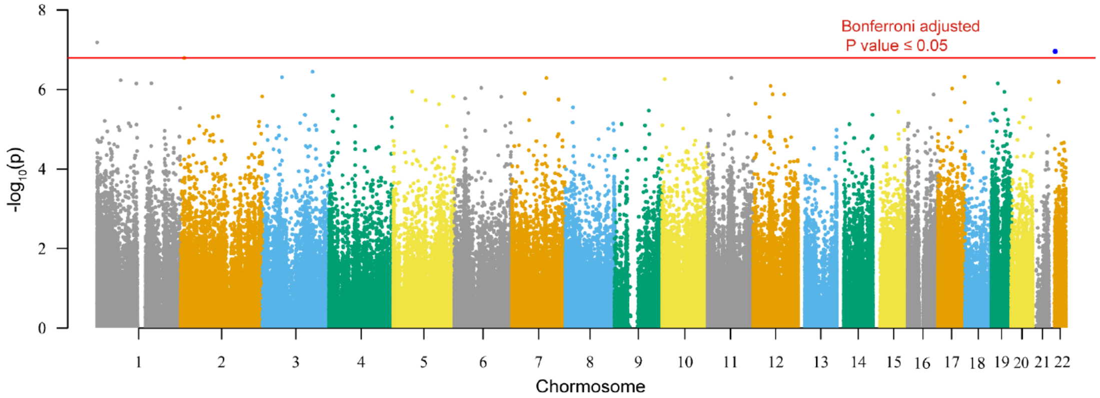

Supplementary Figure 1. Manhattan plot of methylation–age interaction *P*-values derived from Cox proportional hazards model adjusted for age, smoking status, sex, clinical stage, and study cohort in lung adenocarcinoma (LUAD) patients in the discovery phase. Red line represents Bonferroni adjusted $P \le 0.05$. Blue dot represents one CpG probe identified in this study, with Bonferroni adjusted $P \le 0.05$ in the discovery phase, $P \le 0.05$ in the validation phase, and consistent effect direction across both phases.

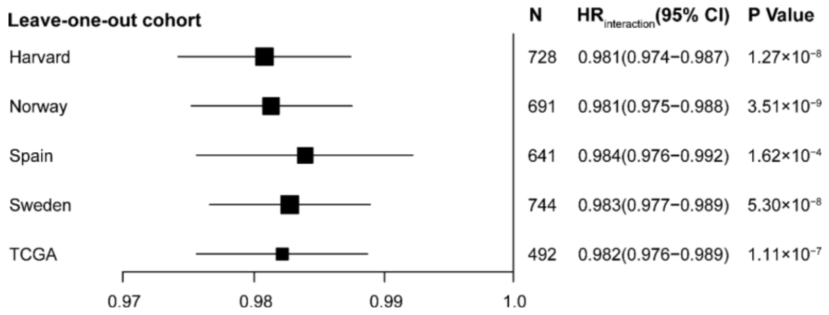

Supplementary Figure 2. Association results of lung adenocarcinoma (LUAD)-specific cg14326354 $_{PRODH}$–age interaction using leave-one-out method for validation. Size of each box represents the sample size of each cohort. Hazard ratio (HR) of cg14326354$_{PRODH}$ 5% per decrement of methylation level and *P*-value of interaction term derived from the Cox proportional hazards model adjusted for age, smoking status, sex, clinical stage, and study cohort in LUAD patients when leaving one cohort out.

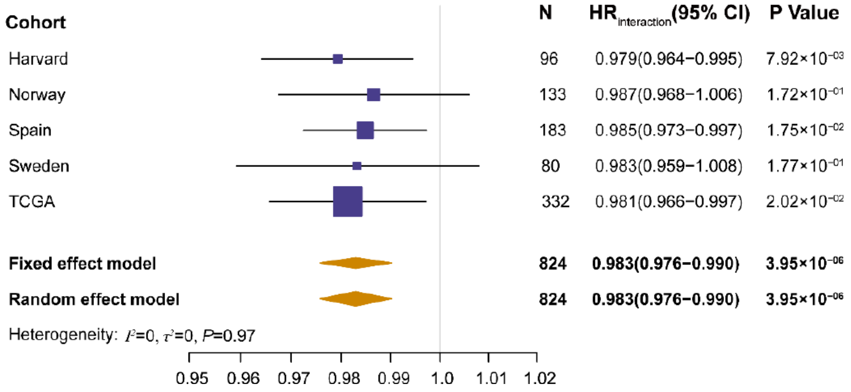

Supplementary Figure 3. Meta-analysis of interaction between DNA methylation of cg14326354$_{PRODH}$ and age for lung adenocarcinoma (LUAD) patients from five cohorts. Fixed effect and random effect models were both applied, and effect heterogeneity among five cohorts was tested. Size of each box represents the sample size of each cohort, and diamond represents the overall effect of cg14326354$_{PRODH}$ in five cohorts. Hazard ratio (HR) of cg14326354$_{PRODH}$ 5% per decrement of methylation level and *P*-value of interaction term derived from the Cox proportional hazards model adjusted for age, smoking status, sex, clinical stage, and study cohort in LUAD patients in each of the five cohorts.

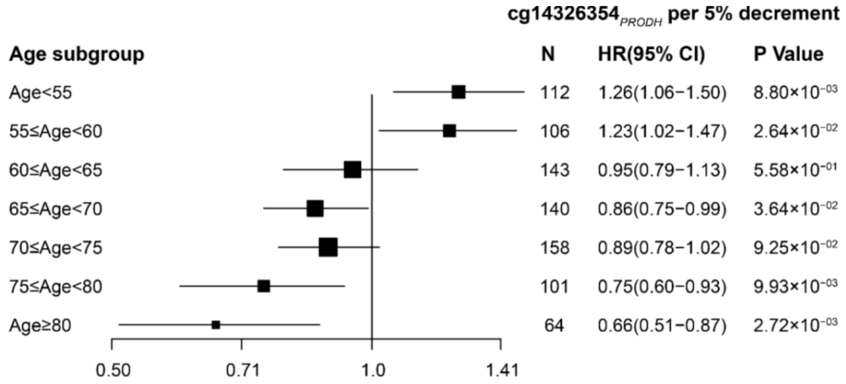

Supplementary Figure 4. Association results of the cg14326354$_{PRODH}$ effect on lung adenocarcinoma (LUAD) survival in different age subgroups. Size of each box represents the sample size of each cohort. Hazard ratio (HR) of cg14326354$_{PRODH}$ 5% per decrement of methylation level and *P*-value derived from the Cox proportional hazards model adjusted for age, smoking status, sex, clinical stage, and study cohort in LUAD patients in each age subgroup.

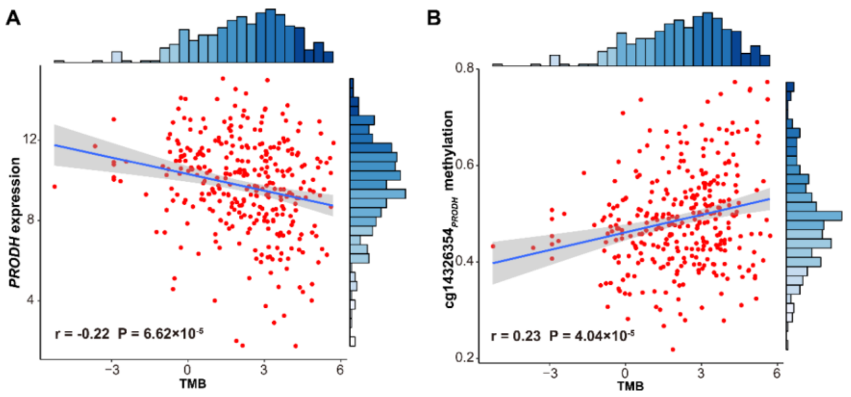

Supplementary Figure 5. Results of correlation analysis between tumor mutational burden (TMB) and *PRODH* methylation as well as expression. Correlation coefficient (*r*) and *P*-value were derived from Pearson correlation analysis. TMB as well as gene expression were log2-transformed before correlation analysis due to violation of normal distribution assumption before data transformation.

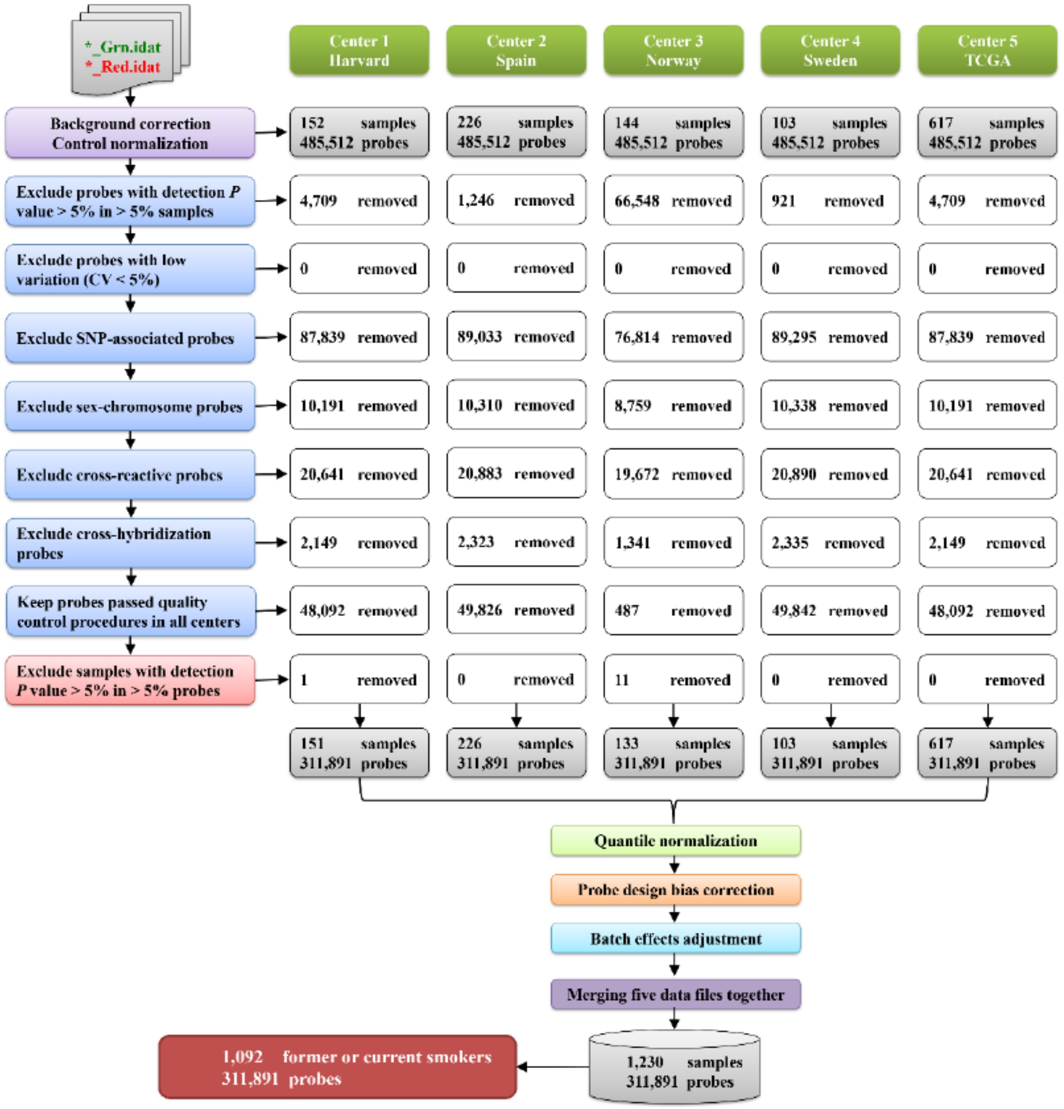

Supplementary Figure 6. Quality control procedures for epigenome-wide DNA methylation data.

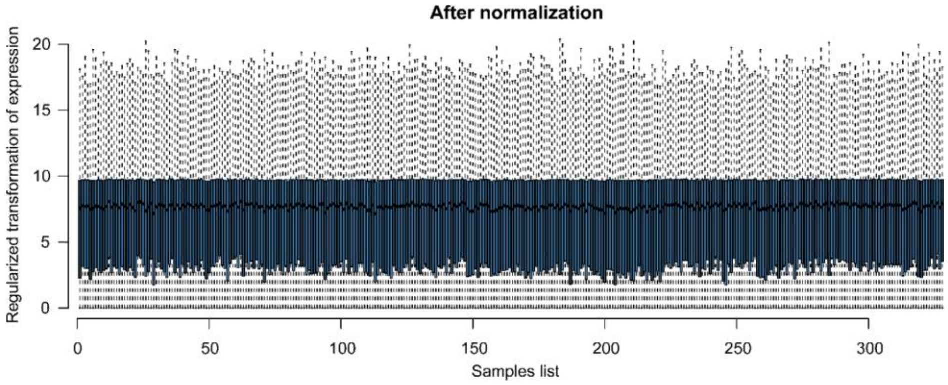

Supplementary Figure 7. Boxplot of gene expression distribution across all samples after normalization. Gene expression values were log_2-transformed.
