# Supplementary material for: Epigenome-wide gene–age interaction analysis reveals reversed effects of PRODH DNA methylation on survival between young and elderly early-stage NSCLC patients
Source: Aging (Albany NY). 2020 Jun 8;12(11):10642–62. doi: 10.18632/aging.103284 (PMC7346054; doi:10.18632/aging.103284)
Supplement: Supplementary Tables [file aging-12-103284-s004..pdf]

SUPPLEMENTARY TABLES

Supplementary Table 1. Demographic and clinical characteristics for early-stage non-small cell lung cancer (NSCLC) patients from five cohorts.

Variable	Discovery phase					Validation phase	Combined dataset
	Cohort 1: Harvard (*N* = 151)	Cohort 2: Spain[a] (*N* = 226)	Cohort 3: Norway (*N* = 133)	Cohort 4: Sweden (*N* = 103)	Discovery: All (*N* = 613)	Cohort 5: TCGA (*N* = 617)	Overall samples (*N* = 1230)
Age (years)	67.67±9.92	65.67±10.58	65.52±9.34	67.54±9.99	66.44±10.08	66.51±9.47	66.48±9.78
Sex, *n* (%)							
Female	67 (44.37)	105 (46.46)	71 (53.38)	54 (52.43)	297 (48.45)	255 (41.33)	552 (44.88)
Male	84 (55.63)	121 (53.54)	62 (46.62)	49 (47.57)	316 (51.55)	362 (58.67)	678 (55.12)
Smoking status, *n* (%)							
Never	18 (11.92)	30 (13.57)	17 (12.78)	18 (17.48)	83 (13.65)	55 (9.18)	138(11.22)
Former	81 (53.64)	120 (54.30)	74 (55.64)	54 (52.43)	329 (54.11)	376 (62.77)	705 (58.41)
Current	52 (34.44)	71 (32.13)	42 (31.58)	31 (30.10)	196 (32.24)	168 (28.05)	364 (30.16)
Unknown	0	5	0	0	5	18	23
Clinical stage, *n* (%)							
I	104 (68.87)	183 (80.97)	93 (69.92)	95 (92.23)	475 (77.49)	393 (63.70)	868 (70.57)
II	47 (31.13)	43 (19.03)	40 (30.08)	8 (7.77)	138 (22.51)	224 (36.30)	362 (29.43)
Histology, *n* (%)							
LUAD	96 (63.58)	183 (80.97)	133 (100.00)	80 (77.67)	492 (80.26)	332 (53.81)	824 (66.99)
LUSC	55 (36.42)	43 (19.03)	0 (0.00)	23 (22.33)	121 (19.74)	285 (46.19)	406 (33.01)
Chemotherapy, *n* (%)							
No	142 (94.04)	177 (90.77)	102 (76.69)	67 (90.54)	488 (88.25)	194 (76.98)	682 (84.72)
Yes	9 (5.96)	18 (9.23)	31 (23.31)	7 (9.46)	65 (11.75)	58 (23.02)	123 (15.28)
Unknown	0	31	0	29	60	365	425
Radiotherapy, *n* (%)							
No	132 (87.42)	184 (94.36)	132 (99.25)	74 (100.00)	522 (94.39)	239 (94.84)	761 (94.53)
Yes	19 (12.58)	11 (5.64)	1 (0.75)	0 (0.00)	31 (5.61)	13 (5.16)	44 (5.47)
Unknown	0	31	0	29	60	365	425
Adjuvant therapy[b], *n* (%)							
No	127 (84.11)	168 (86.15)	101 (75.94)	67 (90.54)	463 (83.73)	187 (74.21)	650 (80.75)
Yes	24 (15.89)	27 (13.85)	32 (24.06)	7 (9.46)	90 (16.27)	65 (25.79)	155 (19.25)
Unknown	0	31	0	29	60	365	425
Survival year							
Median (95% CI)	6.66 (5.41-7.87)	7.12 (5.06-9.63)	7.36 (6.77-7.95)[*]	7.39 (4.98-9.12)	7.39 (6.50-8.23)	4.54 (3.68-5.41)	6.60 (5.84-7.35)
Dead (%)	122 (80.79)	101 (44.69)	42 (31.58)	58 (31.58)	323 (52.69)	142 (23.01)	465 (37.80)

[a]Cohort 2: Spain is a collaborative cohort, recruiting samples from Spain, Italy, UK, France, and USA.
[b]Adjuvant therapy includes chemotherapy or radiotherapy.
[*]Restricted mean survival time is given since median is not available.
LUAD: lung adenocarcinoma; LUSC: lung squamous cell carcinoma

Supplementary Table 2. Results for two lung adenocarcinoma (LUAD)-specific methylation–age interactions identified from a two-stage epigenome-wide association study.

Variable	Discovery phase				Validation phase				Combined data			
	HR	95% CI		*P*	HR	95% CI		*P*	HR	95% CI		*P*
cg14326354	2.986	1.921	4.640	1.16×10^{-06}	3.442	1.146	10.337	2.76×10^{-02}	1.885	1.429	2.487	7.16×10^{-06}
Age	0.860	0.809	0.915	1.74×10^{-06}	0.854	0.728	1.002	5.36×10^{-02}	0.926	0.890	0.964	1.53×10^{-04}
Interaction	0.982	0.976	0.989	1.11×10^{-07}	0.981	0.966	0.997	2.02×10^{-02}	0.989	0.986	0.994	9.18×10^{-07}
cg08700284	62.927	15.807	250.507	4.20×10^{-09}	3.336	0.024	455.52	6.31×10^{-01}	8.960	3.779	21.243	7.16×10^{-06}
Age	0.349	0.237	0.513	9.31×10^{-08}	0.718	0.175	2.947	6.46×10^{-01}	0.591	0.466	0.751	1.62×10^{-05}
Interaction	0.944	0.925	0.964	6.55×10^{-08}	0.981	0.910	1.057	6.17×10^{-01}	0.971	0.959	0.984	9.18×10^{-07}

HR: hazard ratio; 95% CI: 95% confidence interval

Supplementary Table 3. Annotation information for significant lung adenocarcinoma (LUAD)-specific CpG probe.

CpG probe	CHR	BP	Region	Relation to CpG islands	Gene description[a]
cg14326354	22	18900453	3'UTR	S_Shelf	proline dehydrogenase 1 (*PRODH*)

[a]Hyperlinks provide literature-based evidence for each gene from DAVID (https://david.ncifcrf.gov).
CHR: chromosome; BP: basepair

Supplementary Table 4. Results of interaction for sensitivity analysis of one significant lung adenocarcinoma (LUAD)-specific CpG probe.

Variable	Discovery phase				Validation phase				Combined data			
	HR	95% CI		*P*	HR	95% CI		*P*	HR	95% CI		*P*
cg14326354	3.056	1.950	4.792	1.11×10^{-06}	3.522	1.156	10.73	2.67×10^{-02}	3.048	2.043	4.547	4.77×10^{-08}
Age	0.858	0.806	0.914	1.61×10^{-06}	0.853	0.726	1.003	5.36×10^{-02}	0.861	0.814	0.911	1.80×10^{-07}
Interaction	0.982	0.976	0.989	1.03×10^{-07}	0.981	0.966	0.997	2.09×10^{-02}	0.982	0.976	0.988	3.18×10^{-09}

In sensitivity analysis, patients were excluded if their methylation values were out of range mean± 3×standard deviation on $logit_2$ transformed scale.
HR: hazard ratio; 95% CI: 95% confidence interval

Supplementary Table 5. Results of heterogeneity test of the interaction effect between subgroups categorized by covariates.

Covariate	Subgroup	$HR_{interaction}$	95% CI		$P_{interaction}$	$Q_{heterogeneity}$	$P_{heterogeneity}$
Smoking status	Never or former smoker	0.982	0.974	0.989	8.42×10^{-7}	<0.01	0.9928
	Current smoker	0.982	0.971	0.992	7.85×10^{-4}		
Sex	Male	0.983	0.974	0.990	1.57×10^{-5}	0.45	0.5006
	Female	0.978	0.968	0.988	2.10×10^{-5}		
Clinical stage	I	0.985	0.978	0.992	9.56×10^{-5}	1.51	0.2185
	II	0.977	0.965	0.988	1.65×10^{-4}		
Study cohort	Harvard	0.979	0.964	0.995	7.92×10^{-3}	0.49	0.9745
	Norway	0.987	0.968	1.006	1.72×10^{-1}		
	Spain	0.985	0.973	0.997	1.75×10^{-2}		
	Sweden	0.983	0.959	1.008	1.77×10^{-1}		
	TCGA	0.981	0.966	0.997	2.02×10^{-2}		

HR: hazard ratio; 95% CI: 95% confidence interval

Supplementary Table 6. Results of low cg14326354$_{PRODH}$ methylation effect on lung adenocarcinoma (LUAD) survival in young and elderly populations defined using boundary of 95% confidence interval (BoCI) standard.

Population	Discovery phase				Validation phase				Combined data			
	HR	95% CI		*P*	HR	95% CI		*P*	HR	95% CI		*P*
Young (age <57 years)	1.182	1.005	1.389	4.29×10^{-2}	1.294	1.023	1.636	3.18×10^{-2}	1.200	1.030	1.401	1.97×10^{-2}
Elderly (age >65 years)	0.810	0.742	0.885	3.12×10^{-6}	0.858	0.754	0.976	1.99×10^{-2}	0.814	0.751	0.882	5.38×10^{-7}

Patients from Harvard, Spain, Norway, and Sweden cohorts were assigned to discovery phase; patients in TCGA were assigned to validation phase.
HR: hazard ratio; 95% CI: 95% confidence interval

Supplementary Table 7. Correlation analysis of association between cg14326354$_{PRODH}$ methylation and proliferation-associated gene expression in lung adenocarcinoma (LUAD) patients using The Cancer Genome Atlas data, as well as survival analysis of proliferation-associated genes (from KEGG database).

Gene	Correlation analysis				Survival analysis			
	r	95% CI		*P*	HR	95% CI		*P*
BTG2	**-0.313**	**-0.408**	**-0.212**	$\mathbf{6.80\times10^{-09}}$	**0.704**	**0.564**	**0.878**	$\mathbf{1.83\times10^{-03}}$
NPDC1	-0.266	-0.364	-0.163	9.88×10^{-07}	0.937	0.755	1.164	0.558
KIAA1524	**0.243**	**0.139**	**0.342**	$\mathbf{8.40\times10^{-06}}$	**1.383**	**1.103**	**1.733**	$\mathbf{4.94\times10^{-03}}$
MKI67	**0.199**	**0.093**	**0.301**	$\mathbf{2.84\times10^{-04}}$	**1.440**	**1.115**	**1.859**	$\mathbf{5.14\times10^{-03}}$
BTG1	-0.188	-0.290	-0.081	6.29×10^{-04}	1.257	0.869	1.819	0.225
BOP1	0.122	0.014	0.227	2.72×10^{-02}	1.170	0.910	1.505	0.222
BTG4	-0.118	-0.223	-0.010	3.29×10^{-02}	0.995	0.769	1.288	0.971
CDC123	**0.111**	**0.003**	**0.217**	$\mathbf{4.46\times10^{-02}}$	**1.798**	**1.045**	**3.097**	$\mathbf{3.41\times10^{-02}}$
PA2G4	0.091	-0.017	0.197	9.94×10^{-02}				
MTCP1NB	0.079	-0.030	0.185	1.55×10^{-01}				
C8orf22	-0.067	-0.174	0.042	2.29×10^{-01}				
HEY1	0.061	-0.048	0.168	2.71×10^{-01}				
PPDPF	-0.058	-0.166	0.050	2.92×10^{-01}				
SIPA1L2	0.055	-0.054	0.162	3.24×10^{-01}				
MTCP1	0.047	-0.061	0.155	3.94×10^{-01}				
SIPA1L3	-0.039	-0.146	0.070	4.84×10^{-01}				
URGCP	0.036	-0.073	0.144	5.17×10^{-01}				
SAV1	-0.032	-0.139	0.077	5.69×10^{-01}				
BTG3	-0.024	-0.132	0.084	6.63×10^{-01}				
PDS5B	-0.023	-0.131	0.085	6.74×10^{-01}				
SIPA1	-0.008	-0.117	0.100	8.80×10^{-01}				
PEA15	-0.006	-0.114	0.103	9.20×10^{-01}				
SIPA1L1	0.000	-0.108	0.108	9.99×10^{-01}				

Correlation coefficient (*r*), 95% CI, and *P*-values were derived from Pearson correlation analysis; survival analysis HR, 95% CI, and P-values were derived from Cox proportional hazards model.
HR: hazard ratio; 95% CI: 95% confidence interval.

Supplementary Table 8. Results of heterogeneity test of the interaction effect between lung adenocarcinoma (LUAD) and lung squamous cell carcinoma (LUSC) populations.

CpG probe	LUAD			LUSC			Heterogeneity	
	HR	95% CI		HR	95% CI		*Q*	*P*
cg08470135	0.944	0.925	0.964	0.975	0.946	1.004	2.97	0.0847
cg14326354	0.982	0.976	0.989	0.982	0.969	0.995	0.00	0.9764

HR: hazard ratio; 95% CI: 95% confidence interval.
